# How I treat: Diagnostic clues and treatment for activated phosphoinositide 3-kinase delta syndrome

**DOI:** 10.70962/jhi.20260054

**Published:** 2026-07-27

**Authors:** Hirokazu Kanegane, Dan Tomomasa, Satoshi Okada

**Affiliations:** 1 Department of Child Health and Development, Graduate School of Medical and Dental Sciences, Institute of Science Tokyo, Tokyo, Japan; 2 Department of Pediatrics and Developmental Biology, Graduate School of Medical and Dental Sciences, Institute of Science Tokyo, Tokyo, Japan; 3 Department of Pediatrics, Hiroshima University Graduate School of Medicine and Health Sciences, Hiroshima, Japan

## Abstract

Activated phosphoinositide 3-kinase delta syndrome (APDS) is a form of common variable immunodeficiency (CVID) caused by gain-of-function variants in *PIK3CD* (APDS1) or loss-of-function variants in *PIK3R1* (APDS2), resulting in hyperactivation of the PI3Kδ pathway. Clinically, it is characterized by recurrent respiratory infections, lymphadenopathy, splenomegaly, and progressive airway destruction, often leading to bronchiectasis. Enteropathy, cytopenia, and immune dysregulation have also been observed in several patients. Some patients develop malignant lymphomas, which can be fatal. Prophylactic antimicrobial therapy and regular immunoglobulin replacement therapy are administered in a manner similar to that used for CVID. However, since excessive activation of the class IA phosphoinositide 3-kinase signaling pathway is the primary pathophysiology of APDS, mammalian target of rapamycin inhibitors and selective p110δ inhibitors are expected to be licensed therapies. Early diagnosis based on diagnostic clues specific to APDS, followed by prompt treatment, is expected to improve patient prognosis.

## Teaser

APDS is a rare inborn error of immunity classified as a group of combined immunodeficiency; however, because specific treatments—such as rapamycin and p110δ inhibitors—for APDS are available, early diagnosis and therapeutic intervention are crucial for improving patient prognosis.

## Introduction

Activated phosphoinositide 3-kinase delta syndrome (APDS) is an inborn error of immunity (IEI) caused by gain-of-function variants in the *PIK3CD* gene, which encodes p110δ, the catalytic subunit of class I phosphoinositide 3-kinase delta (PI3Kδ), resulting in APDS1 (1, 2), or by variants in *PIK3R1,* which encodes p85α, the regulatory subunit of PI3Kδ, resulting in APDS2 (3, 4). Both subunits are expressed mainly in hematopoietic cells. Both types of APDS result in excessive accumulation of phosphatidylinositol 3,4,5-trisphosphate (PIP3) due to constitutive activation of class I PI3Kδ, which leads to overactivation of the PI3Kδ signaling pathway and causes hyperphosphorylation of downstream Akt/mammalian target of rapamycin (mTOR)/S6 molecules. Akt is a key molecule that regulates cell proliferation, differentiation, growth, and metabolism. Hyperphosphorylation results in lymphocyte hyperactivation and lymphoid tissue proliferation (5). Furthermore, loss-of-function variants of phosphatase and tensin homolog (*PTEN)* have been identified in six patients presenting with APDS-like symptoms (6, 7, 8). PTEN catalyzes the dephosphorylation of PIP3, thereby suppressing its expression and inhibiting signaling via the Akt/mTOR/S6 pathway. Thus, *PTEN* loss-of-function variants can result in diseases such as APDS. Immunodeficiency due to *PTEN* abnormalities is termed APDS-like immunodeficiency.

Similar to common variable immunodeficiency (CVID), APDS presents with immune deficiency or hypogammaglobulinemia, resulting in recurrent severe respiratory tract infections (RTIs). However, it has features that are distinct from typical CVID, including the development of bronchiectasis, which often begins in early childhood. Other findings include lymphadenopathy (lymphoid hyperplasia), which is frequently observed in both the gastrointestinal and respiratory systems. Susceptibility to Epstein-Barr virus (EBV) and cytomegalovirus (CMV), along with lymphadenopathy, significantly increases the likelihood of lymphoid malignancy, with diffuse large B cell lymphoma being the most common. Laboratory findings vary widely, ranging from immunoglobulin levels within the normal range to hypogammaglobulinemia. However, in some patients, a characteristic lymphocyte profile such as the one shown below may be observed, which serves as a strong diagnostic clue. Immune dysregulation results in a characteristic immature B cell immune phenotype with low levels of naïve B cells, high levels of transitional B cells, and a hyper IgM phenotype. Within the T cell compartment, there is an elevated senescent CD57^+^ T cell population or an exhausted PD-1 T cell population.

**Figure 1.**
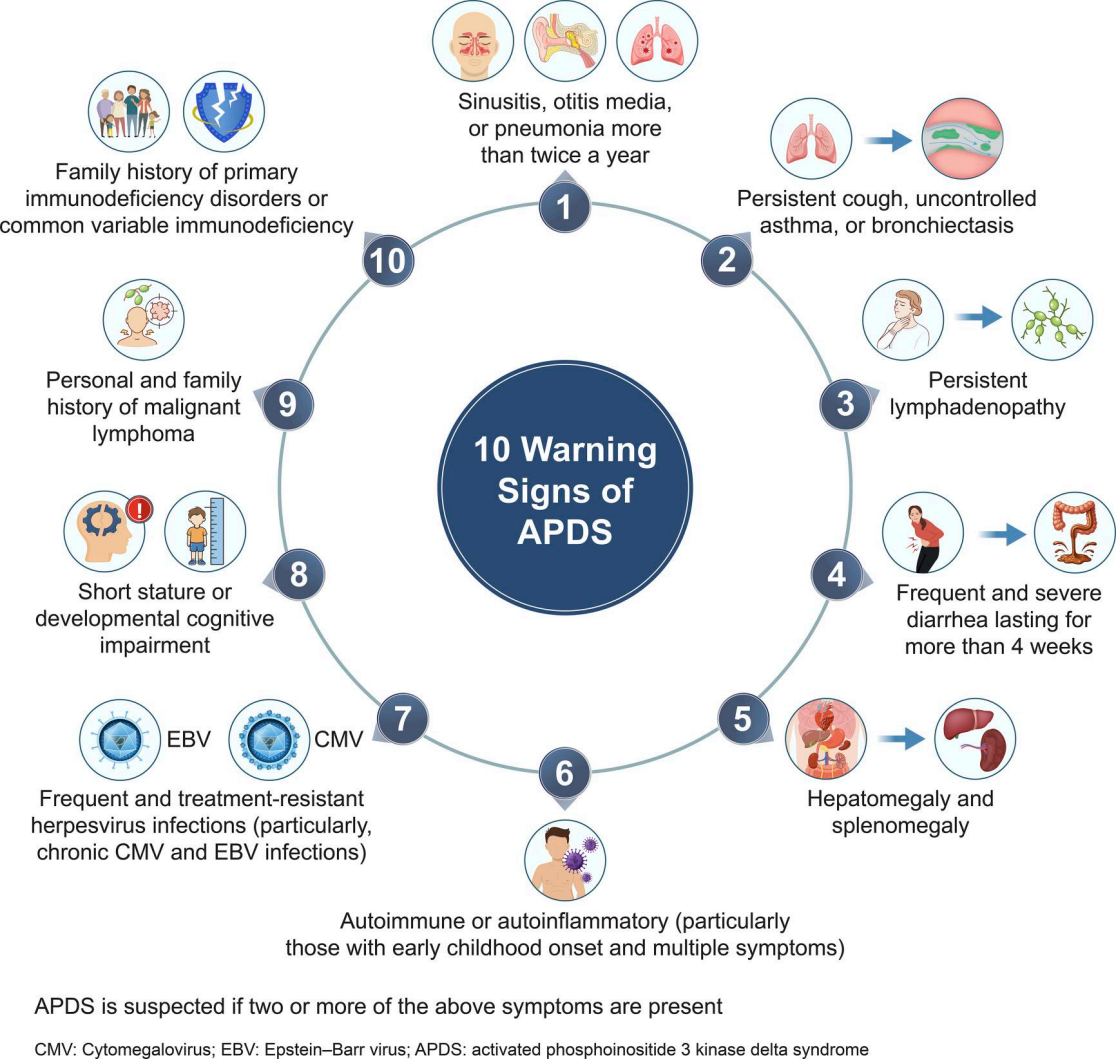
**10 warning signs of APDS.** APDS is suspected if two or more of the symptoms are present.

More than 90% of APDS patients experience recurrent infections (9). Among these patients, those with hypogammaglobulinemia are candidates for immunoglobulin replacement therapy (IgRT). Treatment of APDS involves regular IgRT and prophylactic antibiotic therapy, such as trimethoprim/sulfamethoxazole, for susceptibility to infections due to impaired antibody production, similar to CVID. In cases involving complications of symptomatic herpes virus infections (such as recurrent shingles or herpes labialis), prophylactic oral antiviral medication is considered effective. Patients with severe T cell dysfunction, malignant lymphoma, or autoimmune diseases are candidates for allogeneic hematopoietic cell transplantation (HCT) (10, 11, 12). Corticosteroids and immunosuppressive therapy may also be necessary for associated autoimmune manifestations. Patients with APDS frequently develop lymphoproliferative disorders such as lymphadenopathy and hepatosplenomegaly; reports indicate that mTOR inhibitors (rapamycin) and selective p110δ inhibitors (leniolisib) are effective against these conditions (13, 14, 15). However, their use is currently restricted to certain countries and regions. 17 of the 22 APDS patients who developed malignant lymphoma had previously been diagnosed with benign lymphoproliferative disorders (16). Malignant lymphoma associated with APDS may develop from lymphoproliferative disorders, and suppressing lymphoproliferative disorders may help prevent the onset of malignant lymphoma. However, in very rare cases, malignant lymphoma may develop even during treatment with leniolisib (unpublished data). These cases occurred immediately after leniolisib administration and may be due to the presence of clonal cell populations that had already formed prior to the start of treatment. Long-term follow-up studies of APDS patients treated with leniolisib are needed to determine whether leniolisib actually reduces the risk of developing malignant lymphoma. Although APDS is a very rare IEI, specific treatments, such as selective p110δ inhibitors, particularly leniolisib, are licensed for the treatment of APDS in the US, UK, Australia, Israel, and, most recently, Japan. Therefore, early diagnosis and prompt therapeutic intervention with these agents are expected to improve patient prognosis. The Jeffrey Modell Foundation (https://www.info4pi.org) developed the “10 Warning Signs of Primary Immunodeficiency” to promote the diagnosis of primary immunodeficiency (PID)/IEI as early as possible. Based on these 10 warning signs, we propose the “10 Warning Signs of APDS” for the early clinical detection of APDS (Fig. 1). While the usefulness of these 10 signs remains a topic for future study, this article explains these 10 warning signs to help readers better understand them.

### Sinusitis, otitis media, or pneumonia more than twice a year

These symptoms are characteristic of recurrent upper and lower RTIs. They encompass categories 1 (four or more new ear infections within 1 year), 2 (two or more serious sinus infections within 1 year), and 4 (two or more cases of pneumonia within 1 year) in the 10 Warning Signs of PID. Recurrent RTIs are frequently observed in patients with predominantly antibody deficiency. When these symptoms are present, serum immunoglobulin levels should be promptly measured. Patients with APDS often present with low IgG and IgA, but normal to high IgM levels. Even if IgG levels are not extremely low, a marked IgG2 deficiency may be present; this condition may be misdiagnosed as selective IgG2 deficiency. Hyper-IgM syndrome is the most common misdiagnosis (28.5%) at the initial diagnosis of APDS (17). Hyper-IgMemia is more frequently observed in APDS2 patients than in APDS1 patients (17). When encountering patients with unexplained hyper-IgMemia, genetic analyses of *PIK3CD*, *PIK3R1*, and *PTEN* are recommended. When hypogammaglobulinemia is detected, intravenous or subcutaneous IgRT should be promptly initiated to maintain IgG trough levels at or above 700–1,000 mg/dl (18, 19). In an interim analysis of an open-label extension study of leniolisib, there was a statistically significant reduction in the annualized infection rate (−0.351, P = 0.004), and 37% of patients (10 of 27) who received IgRT at the start of the study reduced their immunoglobulin dosage, with six of those patients discontinuing IgRT entirely (20).

### Persistent cough, uncontrolled asthma, or bronchiectasis

**Figure 2.**
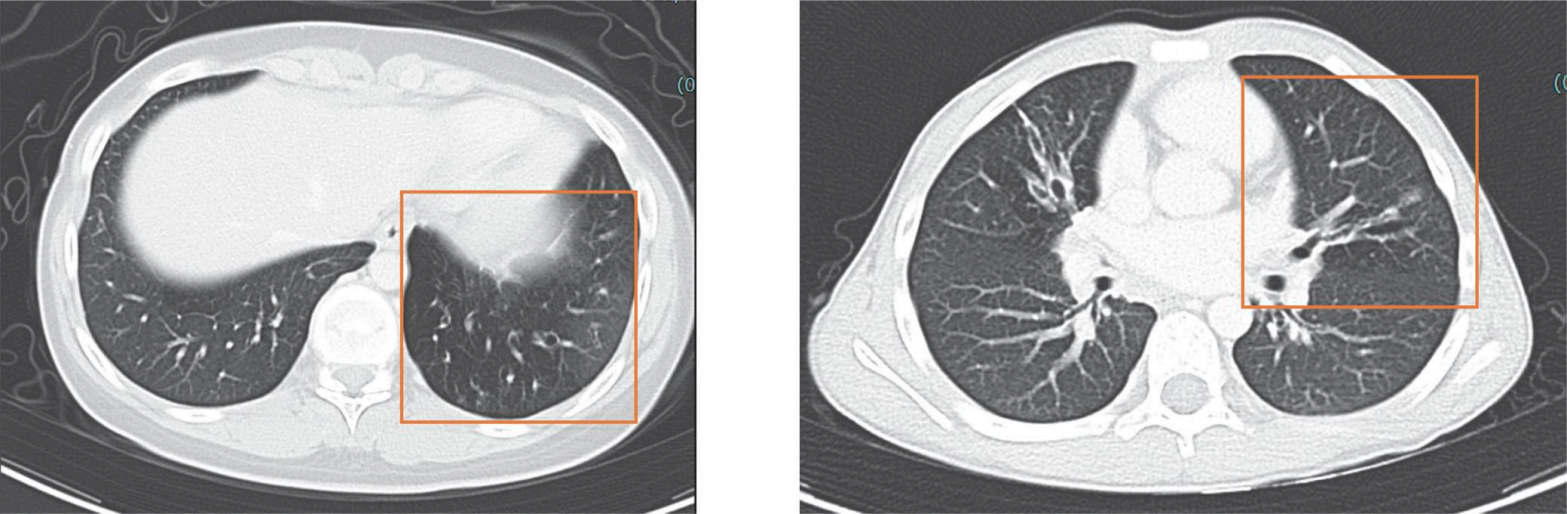
**Chest CT.** Representative axial chest CT images showing bronchiectasis. Bronchiectasis lesions are highlighted in orange boxes. Left and right panels depict patients aged 14 and 10 years, respectively.

When treatment for recurrent lower RTIs is inadequate, chronic bronchitis can develop, presenting with symptoms such as persistent cough and uncontrolled asthma. Bronchiectasis is characterized by irreversible bronchial dilation and damage and remains a major cause of lung injury and healthcare resource utilization in patients with predominantly antibody deficiencies. Bronchial damage is a sequela of recurrent and prolonged inflammatory infections. Chronic lung diseases, such as chronic bronchitis and bronchiectasis, are often seen in adolescence or later in patients with predominantly antibody deficiencies, such as X-linked agammaglobulinemia and CVID (21, 22, 23, 24). However, bronchiectasis can be present in early childhood in patients with APDS (Fig. 2). In a cohort of 143 patients with APDS in Europe, bronchiectasis was observed in 50% of patients at a median age of 7 years (range 1–43 years) (16). Another cohort reported that airway disease associated with bronchiectasis was observed in 52% of patients by the age of 10 and 88% by the age of 40 (25). In APDS, it is not uncommon for bronchiectasis to be present even in patients with childhood-onset disease. It is likely that the mechanism underlying this condition leads to more rapid airway destruction compared with that in other diseases. Bronchiectasis is associated with APDS1 and APDS2 in 60% and 18% of patients, respectively (26, 27). The treatment of bronchiectasis includes antimicrobial prophylaxis to limit ongoing infection and airway clearance techniques to reduce mucus plugging. During a 6-year observation period following leniolisib administration, five of six patients had a history of respiratory disease. Three of the six patients had bronchiectasis at the start of treatment but showed no signs of subsequent disease progression. Pulmonary function tests in all patients revealed no new symptoms or worsening of existing conditions (28).

### Persistent lymphadenopathy

This symptom is characteristic of autoimmune lymphoproliferative syndrome (ALPS) and is rarely observed in typical CVID. Among 39 patients with APDS, one was initially diagnosed with ALPS (17). Hepatosplenomegaly (category 5) and malignant lymphoma (category 9) are also associated with this category. Patients with ALPS frequently present with hepatosplenomegaly and malignant lymphoma. Lymphadenopathy is observed in 64% of patients with APDS1 and 75% of patients with APDS2 (26, 27).

In a phase III placebo-controlled trial of leniolisib in patients with APDS, lymph node size was quantitatively assessed using CT or MRI. The reduction in lymphadenopathy, measured as the change from baseline in the log-transformed sum of the diameters of target lymph nodes at day 85, was −0.27 in the leniolisib group and −0.02 in the placebo group (15). In the safety analysis population, 26% of patients in the leniolisib group (*n* = 19) achieved complete disappearance of major lymphadenopathy, while the remaining 74% achieved a partial response. In the placebo group (*n* = 9), 45% achieved a partial response, 44% had stable disease, and 11% had an unknown response.

In APDS, PI3Kδ is hyperactivating, leading to enhanced downstream mTOR signaling. Rapamycin inhibits this mTOR pathway and is a candidate therapeutic agent for APDS. In a European APDS cohort study by Maccari et al. (13), rapamycin was administered to 26 patients with APDS (17 with APDS1 and 9 with APDS2). According to the physicians’ overall assessment, 19 of the 26 patients (73%) showed moderate or greater improvement. Regarding lymphadenopathy and splenomegaly, 76% achieved complete or partial remission; however, the treatment was ineffective in 64% and 60% of cases for anemia, thrombocytopenia, and enteritis, respectively. Furthermore, rapamycin treatment allowed 7 out of 8 patients taking steroids to discontinue them completely; thus, even when lesions did not achieve complete remission, the benefit of breaking free from steroid dependence was recognized.

### Frequent and severe diarrhea lasting for more than 4 wk

**Figure 3.**
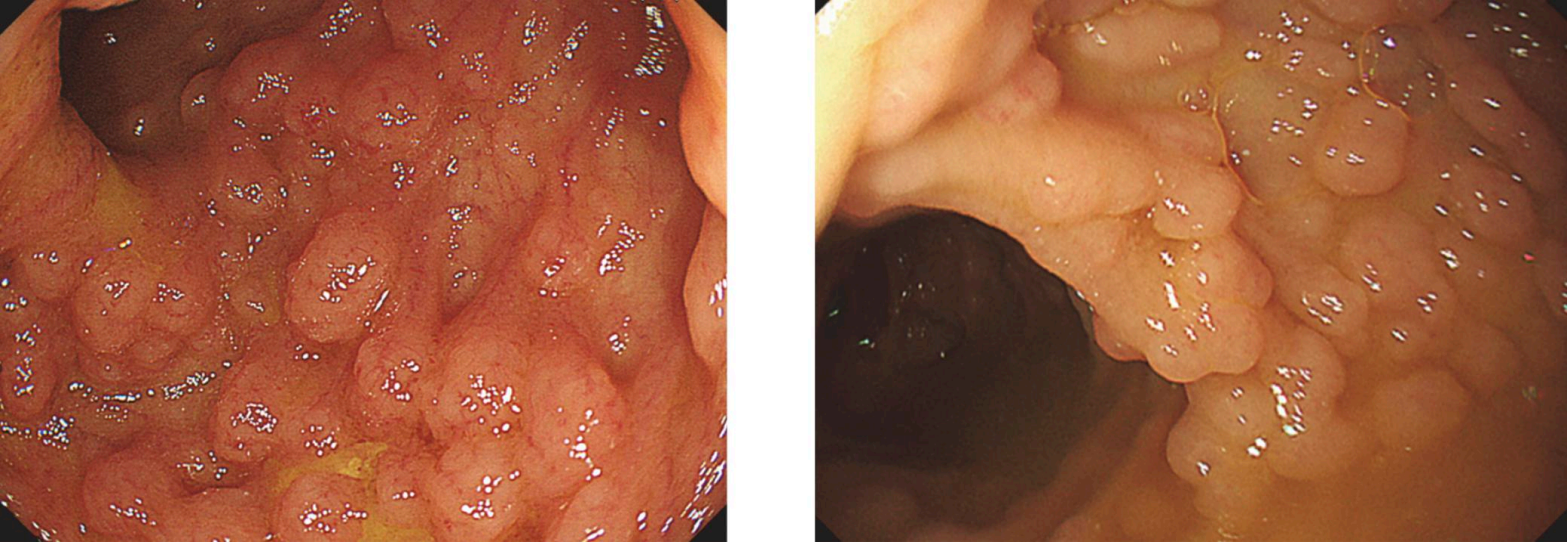
**Endoscopic findings of the intestine.** Representative endoscopic images showing multiple lymphoid follicles in the terminal ileum. Left and right panels depict patients aged 36 and 6 years, respectively.

Systemic lymphoid hyperplasia is common in APDS, and intestinal lymphoid follicular hyperplasia is observed as part of this lymphoid hyperplasia (Fig. 3). Intestinal lymphoid follicular hyperplasia was present in 32% of patients with APDS1 and 24% of patients with APDS2 who presented with chronic diarrhea and malabsorption (26, 27). Enteritis is the initial presenting symptom in 5.3% of patients with APDS (17). Enteropathy was observed in 35% of patients with APDS (16). The presence of intestinal lymphoid follicular hyperplasia promotes hypogammaglobulinemia due to protein loss. Protein-losing gastroenteropathy may sometimes occur in conjunction with CVID, in which case high-dose immunoglobulin replacement therapy is recommended (29). A similar treatment approach is also recommended for APDS. Therefore, adequate IgRT is essential, along with therapeutic intervention using immunosuppressive agents. However, treatment with immunosuppressive agents alone may be insufficient. Rapamycin is effective for lymphoproliferative disorders, but its efficacy for enteritis is unclear (25). Furthermore, complete resolution of intestinal lymphoid follicular hyperplasia has been reported following HCT in some patients (8).

### Hepatomegaly and/or splenomegaly

**Figure 4.**
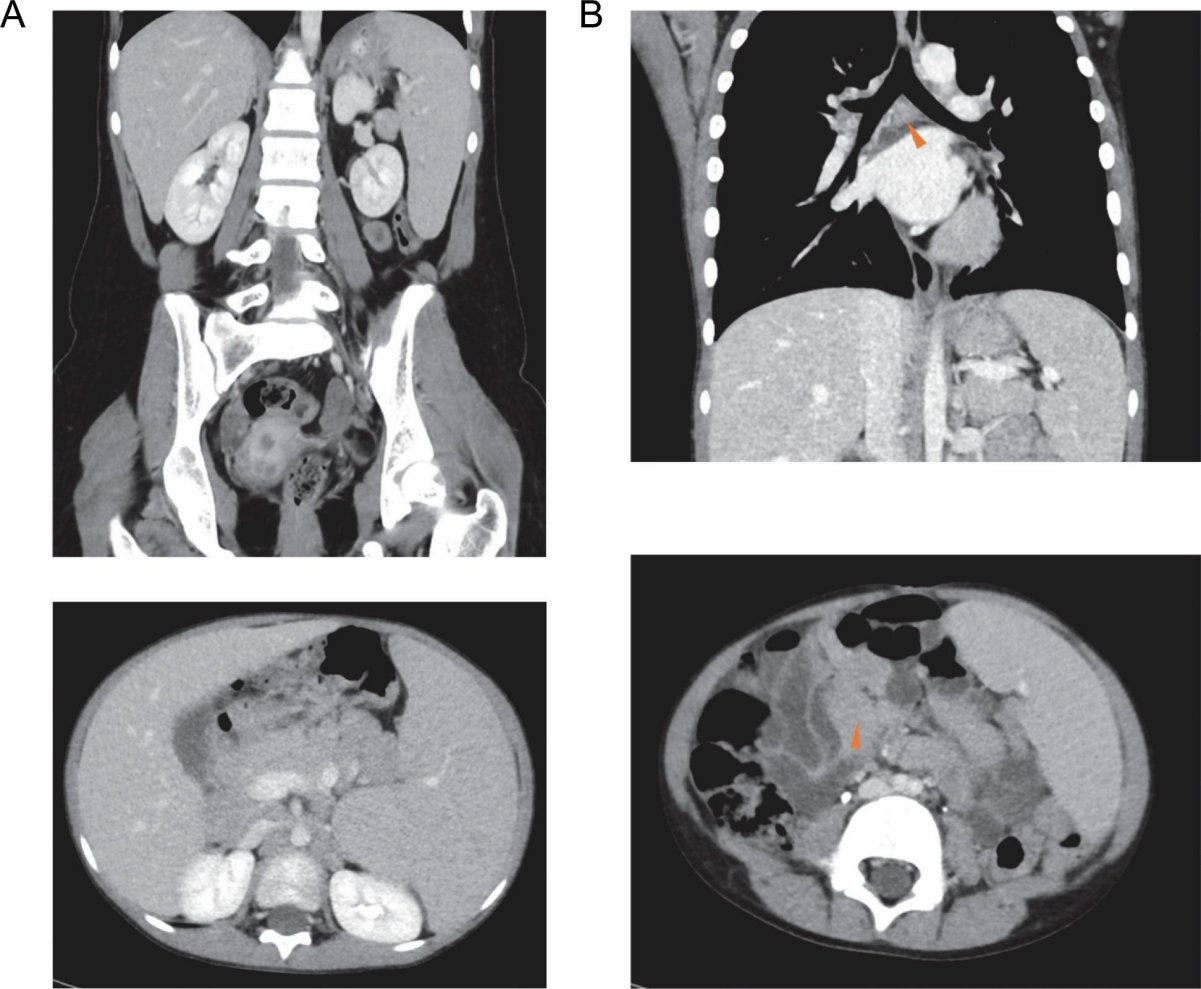
**Abdominal enhanced CT. (A)** Representative images of splenomegaly. Upper and lower panels depict patients aged 14 and 6 years, respectively. **(B)** Representative images of lymphoproliferation, with lesions of interest indicated by orange arrows. Upper and lower panels depict patients aged 10 and 6 years, respectively.

Similar to the lymph nodes, the liver and spleen are involved in lymphoid tissue. Hepatosplenomegaly is a symptom of lymphoid tissue hyperplasia observed in patients with APDS (Fig. 4). Splenomegaly and hepatomegaly were observed in 58% and 45% of patients with APDS1 and APDS2, respectively (26), whereas splenomegaly alone was observed in 43% of patients with APDS2 (27). Since splenomegaly is reported to occur in 15–30% of cases of CVID (30, 31, 32, 33), splenomegaly occurs more frequently in patients with APDS than in those with CVID. Therefore, hepatosplenomegaly in patients with CVID-like symptoms may suggest APDS, particularly APDS1. APDS frequently involves thrombocytopenia, which may result not only from autoimmune mechanisms but also from hypersplenism due to an enlarged spleen.

It is anticipated that the reduction in splenic size following leniolisib administration will increase the platelet count by reducing platelet accumulation within the spleen. In a randomized, placebo-controlled, phase III trial of leniolisib, a comparison between the leniolisib and placebo groups revealed a statistically significant reduction in spleen size, with an adjusted mean difference in three-dimensional volume (15). Administration of leniolisib increased platelet counts. Among patients in the safety analysis population who had splenomegaly at baseline, 38% in the leniolisib group (*n* = 13) achieved a complete response, 54% achieved a partial response, and 8% showed stable disease at week 12. In the placebo group (*n* = 5), 20% achieved a complete response, and the remaining 80% experienced disease progression.

### Autoimmune or autoinflammatory diseases

Class IA PI3Ks, including p110δ and p85α, are located downstream of various surface receptors—such as B cell receptors, T cell receptors, Toll-like receptors, G-protein-coupled receptors, and cytokine receptors—and play a critical role in their signal transduction. Autoimmunity in ADPS is thought to involve both humoral and cellular mechanisms (5). Similar to ALPS, APDS is frequently associated with autoimmune diseases. Autoimmune diseases are present in 42% of patients with APDS1 and 17% of patients with APDS2 (26, 27). Similar to ALPS, autoimmune cytopenias, including immune thrombocytopenia, hemolytic anemia, and Evans syndrome, are commonly observed. Other autoimmune diseases include glomerulonephritis, type 1 diabetes mellitus, autoimmune thyroiditis, and arthritis. Inflammatory brain diseases account for <5% of APDS cases (16). Since the mechanisms underlying susceptibility to infection and the onset of autoimmune diseases in APDS are believed to be different, there is no clear evidence that patients treated with IgGT are more prone to developing autoinflammatory diseases.

### Frequent and treatment-resistant herpesvirus infections

In APDS, susceptibility to infection has been demonstrated not only for EBV and CMV but also for herpes simplex virus (HSV) and varicella-zoster virus (VZV). EBV infection is observed in 26% of patients with APDS1 and 22% of those with APDS2, whereas CMV infection is observed in 15% of patients with APDS1 and 17% of patients with APDS2 (26, 27). HSV and VZV have been detected in 21% of patients with APDS1 (26). Although these infections may be asymptomatic and represent only viremia, careful monitoring is essential. To date, 23 cases of malignant tumors have been reported, and EBV infection was detected in 13 of these cases (57%) (34). In other cohorts, lymphoproliferative disorders preceded lymphoma in 17 of 22 cases, and half of these were reported to be EBV associated (16). Special attention should be paid to EBV infection, as it has been implicated in the development of EBV-positive B cell lymphoma. Although many cases of viremia are low-level and rarely cause clinical problems, PI3K inhibitors carry a risk of viral reactivation or even overt infection; therefore, viral monitoring before and after administration is likely necessary.

### Short stature or developmental cognitive impairment

Global developmental or speech delays were observed in 19% of patients with APDS1 (26). PIK3δ is strongly expressed in the mouse central nervous system, which may contribute to the developmental delays in APDS (35). Stature below −2 standard deviations is observed in 45% of patients with APDS2, but this symptom is not necessarily associated with chronic diarrhea (27). Neurodevelopmental delays, including cognitive impairment and learning disabilities, have also been observed in 31% of patients with APDS2.

SHORT syndrome (Mendelian Inheritance in Man 269880) is a rare multisystem disorder clinically characterized by short stature (S), hyperextensibility of joints, and/or inguinal hernia (H), ocular depression (O), Rieger abnormality (anterior segment ocular dysgenesis, characterized by central iris defects) (R), and tooth eruption delay (T) (36). In 2013, the causative gene for SHORT syndrome was identified as *PIK3R1*, revealing that lipodystrophy and insulin resistance are the primary hallmarks (37, 38, 39). Most *PIK3R1* variants in APDS2 cause exon 11 skipping, whereas *PIK3R1* variants in SHORT syndrome frequently affect the C-terminal Src homology 2 domain. Differences in variant location may lead to distinct interactions with phosphorylated receptor tyrosine kinases, including *PIK3R1*, potentially resulting in different disease phenotypes between APDS2 and SHORT syndrome (40). However, patients with APDS2 exhibiting SHORT syndrome features have also been reported, making differentiation between these two conditions challenging (41, 42, 43). The higher prevalence of short stature and neurodevelopmental delay in APDS2 compared with APDS1 may be due to the ubiquitous expression of the *PIK3R1* gene product, p85α.

### Personal and/or family history of malignant lymphoma

Malignancy is the most serious complication in patients with APDS. Among 243 patients, 31 developed malignancies, with malignant lymphoma being the most frequently observed (89%). Malignant lymphoma occurs in 11% of patients with APDS1 and 28% of patients with APDS2. It is predominantly a B cell lymphoma and may be EBV positive (26, 27). It typically develops at a relatively young age, and the cumulative risk of developing malignant lymphoma by age 40 is reported to be 78% in patients with APDS2 (26). The age at onset of lymphoma in the 13 patients with APDS1 ranged from 18 mo to 27 years, indicating that the age of onset is unpredictable (26). Some patients with APDS and lymphoma also develop other malignancies (16). Cases of malignant lymphoma require chemotherapy and HCT. Of the five reported deaths among 36 patients with APDS2, four were due to malignant lymphoma (26). As malignant lymphoma is thought to develop from lymphoproliferative disorders, suppression of lymphoproliferation by leniolisib may prevent the onset of malignant lymphoma, reduce the need for HCT, and potentially improve patient prognosis (44).

### Family history of PID disorders or CVID

Among the 10 warning signs of PID, category 10 (a family history of PID) is listed, and similar signs have being raised. When diagnosing PID/IEI, family history is considered a more important diagnostic factor than intravenous antibiotic use or growth failure (45). As APDS1 and APDS2 follow an autosomal dominant inheritance pattern, inquiry about family history is crucial; however, many cases result from de novo variants without any family history. It is well known that in IEIs with an autosomal dominant pattern, such as IKAROS deficiency and CTLA-4 haploinsufficiency, de novo cases are common, and there is significant variation in the distribution of symptoms even within the same family (46, 47). It is believed that the same applies to APDS, although no clear data have been reported. Since some patients with APDS show mild or atypical manifestations, careful investigation of their family history and genetic testing are necessary.

## Treatment for APDS

Before the causes and pathophysiology of APDS were understood, it was mainly treated similarly to CVID using IgRT and prophylactic antimicrobial agents. Although these treatments have demonstrated some effectiveness in preventing infections, they are unable to suppress the development of malignant lymphoma. Furthermore, they are not effective in preventing the progression of associated bronchiectasis.

Rapamycin, an mTOR inhibitor, has been used in many patients since the initial report by Lucas et al. (2) and is considered particularly effective against lymphadenopathy and splenomegaly (13). However, it is unclear whether rapamycin administration can prevent the development of malignant lymphoma. Furthermore, its efficacy against infections, autoimmune cytopenias, and enteritis is limited. Long-term administration of mTOR inhibitors is associated with various side effects, including hyperglycemia, dyslipidemia, and cytopenias (48). Since there are no data on the long-term prognosis of rapamycin treatment for APDS, careful follow-up is required.

HCT is the only curative treatment for APDS, including malignant lymphoma. If the transplant is successful, patients are freed from all drug therapies, including IgRT. However, transplant complications such as graft-versus-host disease and posttransplant hemophagocytic lymphohistiocytosis are frequently observed, and transplant-related deaths are sometimes unavoidable (11, 12). According to an international retrospective study (*n* = 57), 8 of the 47 cases that achieved engraftment were in a mixed chimeric state; of these, one case exhibited immune thrombocytopenia and hypogammaglobulinemia, which were believed to be associated with APDS (12).

Leniolisib, a selective PI3Kδ inhibitor, has a more tolerable side effect profile than conventional PI3K inhibitors approved for the treatment of acute lymphoid malignancy and is more effective against APDS because its mode of action directly counteracts the pathophysiology of the condition (49). Thus, leniolisib inhibits lymphoproliferation and the progression of bronchiectasis. Furthermore, by normalizing B cell populations, improving immune competence, and reducing infection burden, it may be possible in patients receiving IgRT to reduce the dosage of immunoglobulin preparations or discontinue IgRT in some cases, making it a groundbreaking therapy. Although the observation period was short, almost no patients with concurrent malignant lymphomas were observed during leniolisib treatment, suggesting that it may also be effective in preventing lymphoma onsets. Since long-term safety has not yet been established and it remains unclear whether it affects short stature or neurodevelopmental impairment, further long-term observation is necessary. Although leniolisib is not a substitute for IgRT or prophylactic antimicrobial therapy, it is considered a potential first-line treatment for APDS.

## Conclusion

Similar to all genetically diagnosed IEIs, APDS should be diagnosed early rather than managed as CVID, especially when specific licensed treatment options, such as leniolisib, are available. Referring to the 10 Warning Signs of APDS and performing immunological testing first in suspected cases is highly recommended. In addition to hypogammaglobulinemia and hyper-IgMemia, peripheral blood flow cytometry should examine CD4^+^ T cells, CD45RA^+^ naive T cells, follicular helper T cells, CD27^+^ memory B cells, and transitional B cells (50). A definitive diagnosis requires genetic analysis of *PIK3CD*, *PIK3R1*, and *PTEN*. Most patients harbor hot spot variants; however, increased phosphorylation of AKT and S6 protein in activated T cells is also useful when a variant of unknown significance has been identified through genetic testing (51).

Early diagnosis of APDS is crucial. While further verification is needed to determine whether 10 warning signs of APDS is clinically useful, we hope that, based on the 10 warning signs of APDS, diagnosis and appropriate treatment will be initiated without delay.
